# *MYC* activation cooperates with *Vhl* and *Ink4a/Arf* loss to induce clear cell renal cell carcinoma

**DOI:** 10.1038/ncomms15770

**Published:** 2017-06-08

**Authors:** Sean T. Bailey, Aleisha M. Smith, Jordan Kardos, Sara E. Wobker, Harper L. Wilson, Bhavani Krishnan, Ryoichi Saito, Hyo Jin Lee, Jing Zhang, Samuel C. Eaton, Lindsay A. Williams, Ujjawal Manocha, Dorien J. Peters, Xinchao Pan, Thomas J. Carroll, Dean W. Felsher, Vonn Walter, Qing Zhang, Joel S. Parker, Jen Jen Yeh, Richard A. Moffitt, Janet Y. Leung, William Y. Kim

**Affiliations:** 1 Lineberger Comprehensive Cancer Center, University of North Carolina at Chapel Hill, Chapel Hill, North Carolina 27599, USA; 2 Department of Genetics, University of North Carolina at Chapel Hill, Chapel Hill, North Carolina 27599, USA; 3 Department of Pathology, University of North Carolina at Chapel Hill, Chapel Hill, North Carolina 27599, USA; 4 Department of Internal Medicine, Chungnam National University School of Medicine, Daejeon 35015, Republic of Korea; 5 Department of Pharmacology, University of North Carolina at Chapel Hill, Chapel Hill, North Carolina 27599, USA; 6 Department of Epidemiology, Gillings School of Global Public Health, University of North Carolina at Chapel Hill, Chapel Hill, North Carolina 27599, USA; 7 Department of Pathology, Leiden University Medical Center, Leiden 2333, The Netherlands; 8 Departments of Internal Medicine and Molecular Biology, UT Southwestern Medical Center, Dallas, Texas 75390, USA; 9 Department of Medicine, Stanford University School of Medicine, Palo Alto, California 94305-5151, USA; 10 Department of Biochemistry and Molecular Biology, Penn State Milton S. Hershey College of Medicine, 500 University Drive, Hershey, Pennsylvania 17033, USA; 11 Department of Medicine, University of North Carolina at Chapel Hill, Chapel Hill, North Carolina 27599, USA

## Abstract

Renal carcinoma is a common and aggressive malignancy whose histopathogenesis is incompletely understood and that is largely resistant to cytotoxic chemotherapy. We present two mouse models of kidney cancer that recapitulate the genomic alterations found in human papillary (pRCC) and clear cell RCC (ccRCC), the most common RCC subtypes. MYC activation results in highly penetrant pRCC tumours (*MYC*), while MYC activation, when combined with *Vhl* and *Cdkn2a* (*Ink4a/Arf*) deletion (*VIM*), produce kidney tumours that approximate human ccRCC. RNAseq of the mouse tumours demonstrate that *MYC* tumours resemble Type 2 pRCC, which are known to harbour MYC activation. Furthermore, *VIM* tumours more closely simulate human ccRCC. Based on their high penetrance, short latency, and histologic fidelity, these models of papillary and clear cell RCC should be significant contributions to the field of kidney cancer research.

## Abstract (web summary)

Renal cell carcinoma (RCC) is a common and aggressive malignancy. Here, the authors generate two mouse models of the most common RCC subtypes: the human papillary RCC through *MYC* activation and clear cell RCC through *MYC* activation combined with *Vhl* and *Cdkn2a* deletion.

Renal cell carcinoma (RCC) is among the most common malignancies, with an estimated 65,000 new cases and 14,000 deaths annually in the United States^1^. RCC can be subclassified into distinct histologic subtypes including clear cell RCC (ccRCC), papillary RCC Types 1 and 2, and chromophobe RCC^2^. Inherited RCC can be caused by germline mutations in multiple genes that are linked to specific histologic subtypes. In particular, *VHL*, *MET*, *FH* and *FLCN* genes are linked to development of the clear cell, papillary Type 1, papillary Type 2 and chromophobe RCC subtypes, respectively^2^,^3^,^4^. Not surprisingly, these genes are often found to be mutated in sporadic cases of RCC as well^2^,^3^,^4^,^5^,^6^.

The genetics of ccRCC have been studied in depth^4^. The von Hippel-Lindau tumour suppressor protein (pVHL) is broadly inactivated (∼80%) in sporadic ccRCC by either mutation or promoter hypermethylation^3^,^7^ and its tumour suppressor activity is dependent on its downregulation of the alpha subunits of the hypoxia-inducible factor (HIFα) family of transcription factors and in particular HIF2α (refs 8, 9, 10, 11). In addition to mutations of *VHL*, whole exome sequencing studies have defined a number of other significantly mutated genes in ccRCC such as *PBRM1*, *SETD2* and *BAP1*, many of which are related to histone modification or nucleosome remodelling^4^,^5^,^12^,^13^. ccRCC has a relatively low mutation burden relative to other solid tumours^14^ but does have characteristic large deletions and gains of chromosomes 3p, 14q and 5q, respectively, as well as more focal gains and losses of 8q24 (harbouring *MYC*) and 9p21 (harbouring *CDKN2A*), respectively^5^,^12^,^15^.

Focal amplification of 8q24 has been demonstrated in ∼15–23% of ccRCC in three independent studies and in all studies the amplicon appears to harbour *MYC*^5^,^12^,^15^. Gain of 8q, as assessed by classic cytogenetics, is associated with a high risk of lymph node and distant metastases and is an independent prognostic factor^16^,^17^. Despite this genetic evidence implicating MYC in ccRCC pathogenesis, the role of MYC in ccRCC is complex. Studies demonstrate that HIF2α can potentiate MYC transcriptional activity and that MYC gene signatures are able to define a subtype of ccRCC, while HIF1α has the potential to oppose MYC transcriptional activity^18^,^19^,^20^.

Herein we describe the development of mouse models of papillary and clear cell RCC, by modelling the genetic events found in human kidney cancer. We uncover that MYC activation is sufficient to generate papillary RCC in mice. Moreover, MYC activation, along with *Ink4a/Arf* (*Cdkn2a*) inactivation cooperates with *Vhl* loss to form clear cell RCC that is occasionally metastatic. These GEM models represent the first tractable models of papillary and clear cell RCC that have a predictable latency with high penetrance. These models of papillary and clear cell RCC should therefore be significant contributions to the field of kidney cancer research.

## Results

### Kidney specific MYC activation results in papillary RCC

**Figure 1:**
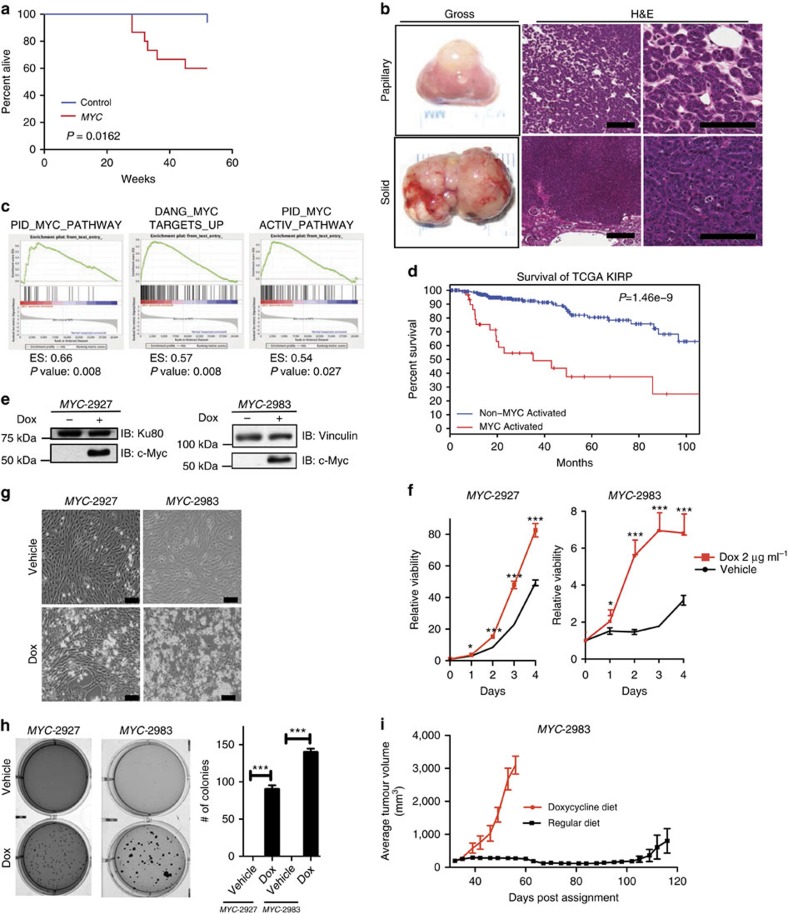
Kidney specific MYC activation results in papillary renal cell carcinoma. (**a**) Kaplan–Meier curve showing reduced survival rates in *MYC* mice (*n*=15) compared to controls (*n*=17) Log Rank *P*=0.0162. (**b**) Representative gross images and photomicrographs of H&E stained kidney sections from *MYC* mice (∼32 weeks post Dox treatment) revealed both papillary (top panels) and solid kidney tumours (bottom panels) (scale bar, 100 μm). (**c**) GSEA enrichment plots showing papillary kidney tumours are enriched for multiple gene sets representing MYC activation relative to normal kidney (GSE11151). (**d**) Kaplan–Meier curve of patients from the TCGA KIRP dataset demonstrating tumours with high MYC activity have reduced survival in patients with papillary renal cell carcinomas, Log Rank *P*=1.46e–9. (**e**) Immunoblot using whole cell lysates from *MYC* kidney tumor-derived cell lines (*MYC*-2927 and *MYC*-2983) shows expression of MYC is Dox dependent. (**f**) Cell viability assay show reduced proliferation from *MYC*-2927 and *MYC*-2983 cells upon Dox removal. Cells grown on Dox or vehicle were analysed in replicates of *n*=8 each day. (**g**) Bright field image of *MYC*-2927 and *MYC*-2983 cells on day 3 after being cultured with or without Dox. (scale bar, 100 μm). (**h**) Soft agar assays show anchorage independent growth of *MYC*-2927 and *MYC*-2983 cells is significantly reduced upon removal of Dox. Images are representative of each condition performed in triplicate. (**i**) Results from xenograft studies showing tumours formed from *MYC*-2983 cells remain dormant upon removal of Dox *in vivo* (*n*=5 per group). (**f**,**g**) *P<0.05, ***P<0.0001. (**f**,**h**,**i**) Data are presented as mean ±s.e.m. (**f**,**h**) *P* values obtained from student *t*-test.

Prior work has examined the role of MYC in the development of kidney cancer by overexpressing MYC under control of the gamma-glutamyl transferase promoter^21^. While the mice developed renal tumours, they appeared to be histologically and immunophenotypically most consistent with collecting duct carcinomas. We generated compound mutant mice engineered to express a doxycycline-inducible Myc transgene (*Tet-O-MYC*) targeted to the renal tubule cells under control of the Ksp promoter^22^,^23^ (*Ksp-rtTA; Tet-O-MYC* mice; hereafter called ‘*MYC*' mice) and control animals expressing only *Tet-O-MYC*. Mice were fed chow containing doxycycline starting at ∼8 weeks of age to induce *MYC* expression and were followed for survival. *MYC* mice had a significantly shortened survival relative to controls (Fig. 1a, *P*=0.0162) and upon necropsy were found to harbour renal tumours but no evidence of macroscopic metastatic disease (Fig. 1b).

Histologic examination showed that the majority of the tumours found in the kidneys of *MYC* mice had either a papillary or a more solid and infiltrative appearance. Smaller tumours (<3 mm) were predominantly papillary (Fig. 1b ‘papillary'), while larger tumours (>3 mm) were either papillary or consisted of more solid appearing tumours characterized by tightly packed papillary structures lacking distinct fibrovascular cores (Fig. 1b ‘solid'). These larger, solid tumours were characterized by hyperchromatic cells with a high nuclear to cytoplasmic ratio. In addition, the cells contained nuclei with large nucleoli, significant pleomorphism and were high grade (Supplementary Fig. 1A). Notably, intra-tumoral foamy macrophages and psammoma bodies (features that are commonly seen in human papillary renal cell carcinoma) were lacking in the *MYC* tumours.

### Papillary RCC with MYC activation has a worse prognosis

Prior work has shown that a subset of pRCC, primarily Type 2 pRCC, is enriched for gene signatures of MYC activation and that tumours with MYC activation have a worse overall survival^24^. We confirmed and extended these findings using multiple gene expression datasets, which revealed that pRCC tumours were enriched for MYC activation gene signatures when compared to normal kidney (Fig. 1c and Supplementary Fig. 1B)^25^. Finally, to determine the impact of MYC activation on prognosis, we classified TCGA KIRP tumours as MYC activated (*n*=23, defined as tumours with a Z score of the PID_MYC_ACTIV_PATHWAY greater than 1.0 s.d. above the mean) or not MYC-activated (*n*=153). Consistent with previous work, patients with MYC activated pRCC tumours had a significantly worse overall survival (Fig. 1d, *P*=1.46e–9)^24^.

### Cell lines from *MYC* mice are dependent upon MYC expression

To better characterize the phenotype of the kidney tumours from *MYC* mice we generated two cell lines from separate *MYC* tumours (*MYC*-2927 and *MYC*-2983). After verifying the Dox responsiveness of the *Tet-O-Myc* transgene in these *MYC* cells (Fig. 1e), we examined the effect of MYC expression on cell proliferation *in vitro*. *MYC* cells grown in the presence of Dox (activated *MYC*) had increased proliferation at 4 days (Fig. 1f), as well as a visible increase in cell number (Fig. 1g). In addition, while *MYC* cells without Dox were unable to form colonies in soft agar, *MYC* cells grown in Dox formed colonies robustly in an anchorage independent manner (Fig. 1h). Finally, we assessed the ability of *MYC-*2983 cells to form tumours *in vivo* and their *in vivo* growth dependence on MYC expression. To this end, 5 × 10^6^ *MYC-*2983 cells were injected subcutaneously and monitored for growth in mice fed Dox chow. Once tumours reached ∼300 mm^3^, mice were either continued on or withdrawn from Dox (Fig. 1i). As expected, tumours of mice that remained on Dox continued to have rapid growth, while tumours in mice withdrawn from Dox remained dormant. Therefore, *MYC-*induced papillary RCCs are dependent upon *MYC* expression for *in vitro* and *in vivo* growth.

### *Vhl* loss with *MYC* activation promotes clear cell changes

Focal amplification of 8q24 is found in 15% of ccRCC in the TCGA KIRC data set^5^. To ensure that MYC was located in the minimal common region (MCR) of amplification, we used GISTIC 2.0 analysis to identify a broad statistically significant (*q* value<0.25) region of amplification on chr8. This MCR contains MYC. A plot of mean gene-level DNA copy number measurement by genomic position (Supplementary Data 1 and Supplementary Fig. 2A) shows that mean MYC copy number is larger than the 70% of the mean gene-level DNA copy values. Therefore, while these data suggest that *MYC* is an important target of amplification in this region there are still ∼100 genes with mean copy number values that are higher. Similarly, focal loss of 9p21 was found in 32% of ccRCC in the TCGA KIRC data set^5^. GISTIC 2.0 analysis demonstrated the presence of only three genes in the minimal common region of copy number loss, *CDKN2A*, *CDKN2B* and *C9orf53* (Supplementary Fig. 2B). These results suggest that both *MYC* and *CDKN2A* may be involved in the development or progression of ccRCC.

To model the interplay of genomic events observed in human RCC, we next examined the phenotypes of kidney specific *Vhl* inactivation in combination with *MYC* overexpression or combined *MYC* overexpression and *Ink/Arf* deletion. Specifically, *MYC* mice were crossed to conditional *Vhl* knock-out mice (*Vhl*^*F/F*^)^26^, *Cdkn2a* (*Ink4a/Arf*^*−/−*^) germline knock-out mice^27^, as well as mice expressing tamoxifen-inducible Cre recombinase under control of the Ksp cadherin promoter (*KspCre*^*ERT2*^) to generate cohorts of *KspCre*^*ERT2*^*; Vhl*^*F/F*^ control mice (conditional inactivation of *Vhl*, hereafter called *V*), *KspCre*^*ERT2*^*; Vhl*^*F/F*^*; Ksp-rtTA; Tet-O-Myc* (conditional inactivation of *Vhl* and Dox-inducible *MYC* overexpression, hereafter called *VM*), and *KspCre*^*ERT2*^*; Vhl*^*F/F*^*; Ink/Arf*^−/−^*; Ksp-rtTA; Tet-O-Myc* (conditional inactivation of *Vhl*, germline *Ink/Arf*^−/−^, and Dox inducible *MYC* overexpression, hereafter called *VIM*) (see Supplementary Table 1).

**Figure 2:**
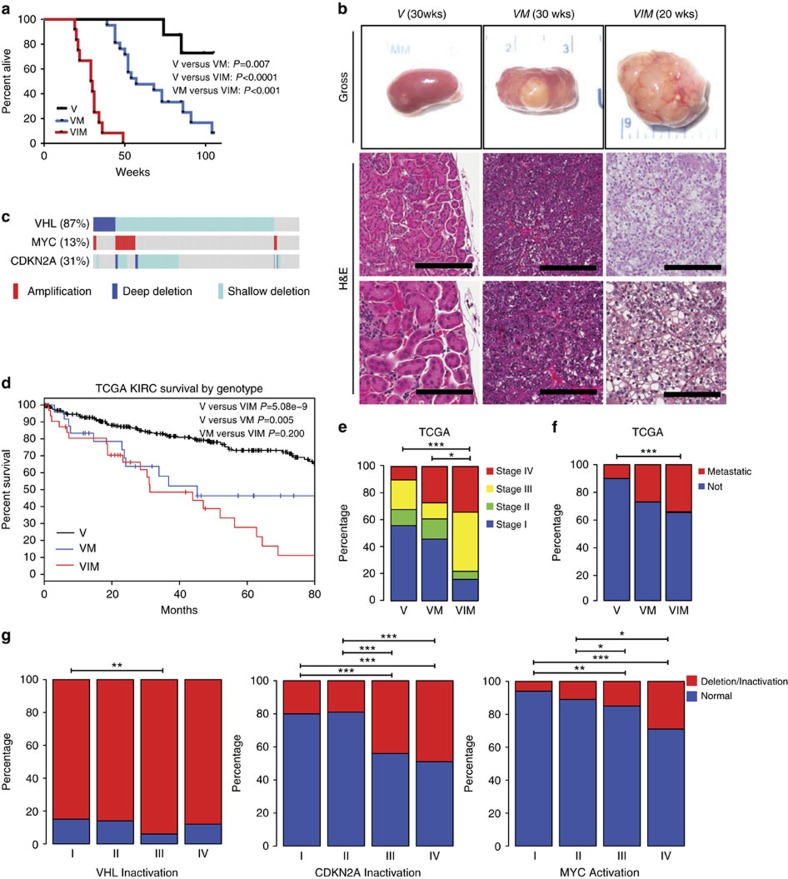
MYC activation combined with *Vhl* and *Ink4a/Arf* loss results in histopathological changes in the kidney resembling human clear cell renal carcinomas. (**a**) Kaplan-Meier survival curve comparing survival rates between *V* (*n*=8), *VM* (*n*=21) and *VIM* (*n*=12) mice. Log rank V versus VM *P*=0.007, V versus VIM *P*<0.0001, VM versus VIM *P*<0.001. (**b**) Representative gross and H&E images of kidney sections from *V* (33 weeks), *VM* (30 weeks) and *VIM* (19 weeks) mice at the indicated times post Dox treatment. (scale bars, 200 and 100 μm). (**c**) cBioPortal OncoPrint plot showing the distribution of *MYC*, *VHL* and *CDKN2A* copy number alterations in the TCGA KIRC data set. (**d**) Kaplan–Meier survival curve comparing human kidney renal clear cell carcinomas with *V*, *VM* and *VIM* alterations. Log rank V versus VIM *P*=5.08e-9, V versus VM *P*=0.005, VM versus VIM *P*=0.200. (**e**) Bar graph showing the correlation of *V*, *VM* and *VIM* tumours from the TCGA KIRC dataset with stage and (**f**) metastasis. (**g**) Bar graphs showing percentage of TCGA KIRC tumours with alterations in VHL, CDKN2A, and MYC by TNM Stage. (**e**–**g**) *P<0.05; **P<0.01, ***P<0.001. *P* values obtained from Chi-square test.

Cohorts of *V*, *VM*, and *VIM* mice were followed for survival (Fig. 2a). *VM* mice had a significantly worse survival than *V* mice (*P*=0.007) and *VIM* mice had a significantly worse survival than both *V* and *VM* mice (both *P*<0.001, Fig. 2a). Histologic examination showed that the kidneys of *V* mice were essentially normal (Fig. 2b). In contrast, kidneys from *VM* and *VIM* mice had a high incidence of tumour formation (67% and 100% respectively, Table 1). Kidney tumours of *VM* mice had either a tubulo-papillary or solid appearance with intervening vessels. The tumour cells were hyperchromatic compared to the normal renal tubular epithelium. Occasional clear cell features were identified (Fig. 2b; Supplementary Fig. 3). Small tumours were generally well demarcated, while larger tumours showed a multilobulated growth pattern with frequent necrosis, haemorrhage and dystrophic calcifications (Supplementary Fig. 4A).

While tumours similar to those seen in the *VM* mice were observed in *VIM* mice, the kidneys of *VIM* mice also harboured tumours with a solid or tightly packed tubulo-papillary appearance. The cells were small to intermediate in size with increased nuclear to cytoplasmic ratio with nuclei that were round to oval with prominent single nucleoli (Supplementary Fig. 3). Clear cell changes within the larger tumours were also present with areas within some tumours showing what appeared to be histology strikingly similar to human ccRCC (Fig. 2b). These areas were typified by nests of tumour cells with an increased amount of clear to granular cytoplasm, separated by thin vascular channels. Therefore, while *Vhl* loss combined with *MYC* activation is sufficient to induce modest clear cell changes, *Vhl* loss combined with *MYC* activation and *Ink/Arf* deletion induces bona fide clear cell RCC (Table 1).

### ccRCC with *VM* and *VIM* alterations have worse prognosis

We next examined whether human ccRCC tumours with similar genomic characteristics (*VHL* inactivation; *VHL* inactivation and *MYC* activation; and *VHL* inactivation, *CDKN2A* loss and *MYC* activation) have similar outcomes as those seen in our mouse models. Of TCGA KIRC tumours (*n*=525), 87, 13 and 31% had *VHL* inactivation, *MYC* activation and *CDKN2A* deletion, respectively (Fig. 2c). We further classified these patients as *V* (*n*=286, 54%), *VM* (*n*=26, 5%) and *VIM* (*n*=32, 6.1%) and noted that *VM* and *VIM* patients had a significantly decreased survival relative to *V* patients (*P*=0.005 and *P*=5.086 × 10^−9^, respectively (Fig. 2d), but that *VM* and *VIM* patients had a similar overall survival. In keeping with the notion that *VIM* tumours are more clinically aggressive, we also noted significant enrichment of higher stage and presence of metastases in patients with *VIM* tumours relative to patients with *V* tumours (Fig. 2e,f). When limiting our analysis to stage IV tumours, we did not see significant survival differences by genotype (*V*, *VM* and *VIM*) (Supplementary Fig. 5). Because of the very limited patient numbers (*V*=28, *VM*=7 and *VIM*=11), we examined whether *VIM* genotype was prognostic when controlled for both TNM stage and Furhman grade. Cox proportional hazards (Cox PH) modelling using the *V* genotype as the reference demonstrated that the *VIM* genotype was still prognostic (Cox PH=1.79, *P*=0.034). Therefore, *VIM* tumours appear to have worse clinical characteristics and outcome in human ccRCC patients even when controlling for stage and grade.

Finally, we attempted to assess whether VHL loss, CDKN2A loss and MYC activation are dysregulated at tumour initiation or tumour progression, by analysing their relative frequency by TNM stage in the TCGA KIRC data set (Fig. 2g). We found that the rate of *VHL* inactivation was relatively similar across all stages, while the rate of *CDKN2A* loss and MYC activation appeared to increase with TNM stage. This data are consistent with the known role of VHL as a gatekeeper tumour suppressor gene and suggests that *CDKN2A* loss and MYC activation are events involved in the progression of a subset of ccRCC tumours.

### *VM* and *VIM* cell lines are dependent upon MYC expression

**Figure 3:**
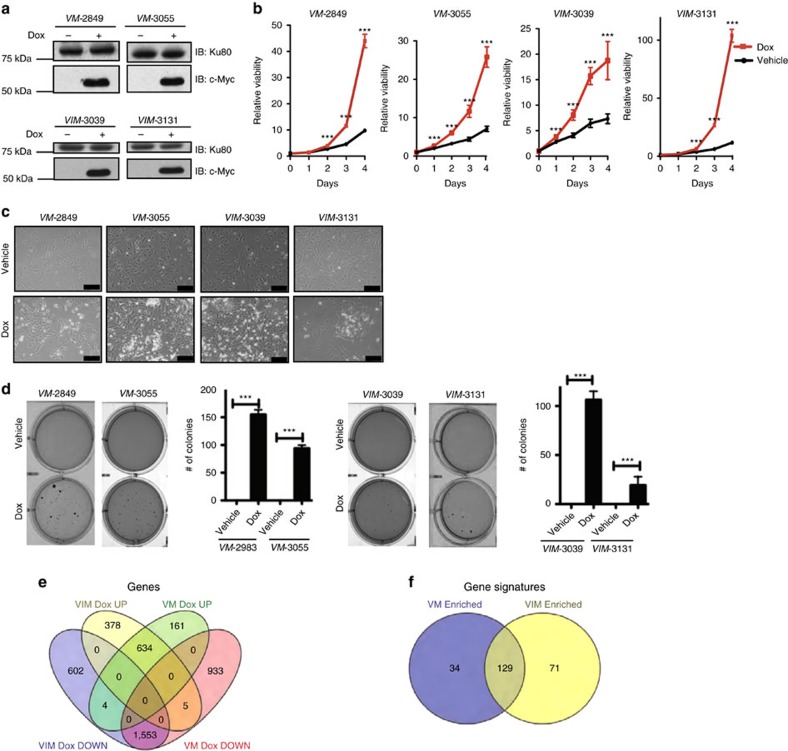
*VM* and *VIM* tumours are dependent on MYC expression. (**a**) Immunoblot of whole cell lysates from *VM* (*VM*-2849 and *VM*-3055) and *VIM* (*VIM*-3039 and *VIM*-3131) tumour-derived cell lines shows expression of MYC is Dox dependent. (**b**) Proliferation of *VM* and *VIM* cells is significantly reduced with removal of Dox. Cells grown on Dox or vehicle were analysed in replicates of *n*=8 each day. (**c**) Bright field image of *VM* and *VIM* cells three days after Dox removal show a decrease in cell number (scale bar, 100 μM). (**d**) Soft agar assays show anchorage independent growth of *VM* and *VIM* cells is reduced upon Dox removal. Images are representative of each condition performed in triplicate. (**e**) Venn diagram showing overlapping and distinct sets of genes altered upon Dox removal in *VM*-3055 and *VIM*-3039 cells. (**f**) Venn diagram showing Dox dependent gene expression in *VM*-3055 and *VIM*-3039 activates distinct gene sets. (**b**,**d**) ***P<0.0001. *P* values obtained from student *t*-test. Data are presented as mean ±s.e.m.

We generated cell lines from *VM* and *VIM* tumours to assess their dependency on MYC expression. We first confirmed the Dox inducibility of the MYC transgene in these *VM* (2849 and 3055) and *VIM* (3039 and 3131) cells (Fig. 3a), as well as the fact that they had undergone Cre mediated recombination of the *Vhl* locus (Supplementary Fig. 4B). Furthermore, we set out to confirm that *VM-3055* and *VIM-3039* cell lines are epithelial in nature. We performed RNAseq and co-clustered mouse 3T3 fibroblasts with *VM-3055* and *VIM-3039* cell lines using the top 10% of the most differentially expressed genes (Supplementary Fig. 4C). 3T3 fibroblasts show a distinctly different expression pattern from both *VM*-3055 and *VIM*-3039 cell lines showing that our *VM* and *VIM* cell lines are not fibroblastic in nature. After confirming that the *VM* and *VIM* cells were derived from renal epithelium, we assessed their growth in *in vitro* assays. Similar to *MYC* cells, the proliferation and anchorage independent growth of *VM* and *VIM* cells were dependent on MYC expression (Fig. 3b–d).

To understand the MYC dependent changes on the transcriptomes of *VM-*3055 and *VIM*-3039 cells, we performed RNAseq on these cells grown in tissue culture in the presence or absence of Dox. There were a large number of genes that were differentially regulated (increased or decreased greater than two-fold; *t*-test FDR<0.05) by *MYC* activation (Dox) in both *VM-*3055 (799 up and 2491 down) and *VIM*-3039 (1017 up and 2159 down) cells (Fig. 3e). While there was overlap among genes that were upregulated by Dox in both *VM-*3055 (634 of 799) and *VIM*-3039 cells (634 of 1017) (Fig. 3e), nearly a third of Dox inducible genes in *VIM*-3039 cells (378 of 1017) were not upregulated in *VM-*3055 cells. A similar percentage of genes repressed by *MYC* activation (Dox) in *VIM*-3039 cells were not repressed in *VM-*3055 cells either, suggesting that MYC may regulate both an overlapping and a unique spectrum of genes in *VM-*3055 and *VIM*-3039 cells.

We next performed gene set analysis. Gene sets that were enriched in *VM-*3055 cells cultured in Dox relative to those cultured without Dox included a number of gene signatures characteristic of MYC activation including those related to cell cycle progression and ribosome biogenesis (Supplementary Data 2). A parallel analysis in *VIM*-3039 cells also demonstrated high enrichment in proliferation and ribosome biogenesis gene signatures (Supplementary Data 3), but also included gene signatures related to DNA methylation, as well as RNA binding. Indeed, the top gene signatures significantly enriched in *VM-*3055 and *VIM*-3039 cells (an area under the curve (AUC) greater than 0.25) were relatively non-overlapping (Fig. 3f). While these findings are in keeping with the notion that MYC activation induces many unique gene expression changes specific to *VM-*3055 and *VIM*-3039 cells given the analysis is on a single-cell line, they will need to be validated in future studies.

### GEM tumours correlate with human RCC

**Figure 4:**
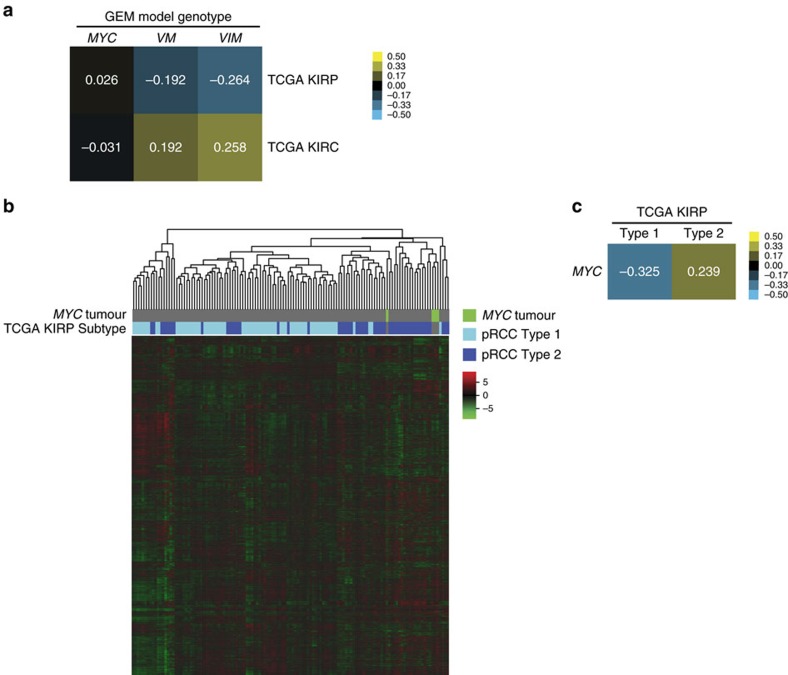
Renal carcinoma mouse models reflect the transcriptomic landscape of human renal carcinoma. (**a**) Pearson correlation of whole-transcriptome centroids of M/VM/VIM mouse models and papillary renal cell carcinoma (KIRP)/clear cell renal cell carcinoma (KIRC) TCGA tumor samples. (**b**) Heatmap clustering of M mouse model samples with TCGA KIRP samples identified by subtype. (**c**) Pearson correlation of whole-transcriptome centroids of the M mouse model with KIRP TCGA Type 1/Type 2 samples.

Given that *MYC* tumours resemble human papillary tumours histologically, while *VM* and *VIM* tumours resemble ccRCC, we wanted to determine if the similarities would also be apparent at the gene expression level. Pearson correlations on whole transcriptome centroids between *MYC* (*n*=4), *VM* (*n*=4) and *VIM* (*n*=4) mouse tumours and the TCGA KIRC and KIRP data revealed that *VM* and *VIM* tumours correlated most highly with TCGA KIRC tumours, while *MYC* tumours correlated more highly with TCGA KIRP tumours (Fig. 4a). Further verifying the importance of MYC activity in pRCC Type 2, a cross species analysis of mouse *MYC* tumours with the TCGA KIRP data set demonstrated co-clustering of *MYC* tumours with human papillary Type 2 tumours (Fig. 4b). Moreover, transcriptome wide Pearson correlations revealed that *MYC* tumours more closely resemble TCGA KIRP tumours verified to be Type 2 pRCC (Fig. 4c). Collectively, these analyses support the clinical relevance of the *MYC*, *VM* and *VIM* mouse models with human papillary and clear cell renal cancers.

### *Ink4a/Arf* loss in *VM* mice promotes metastases

**Figure 5:**
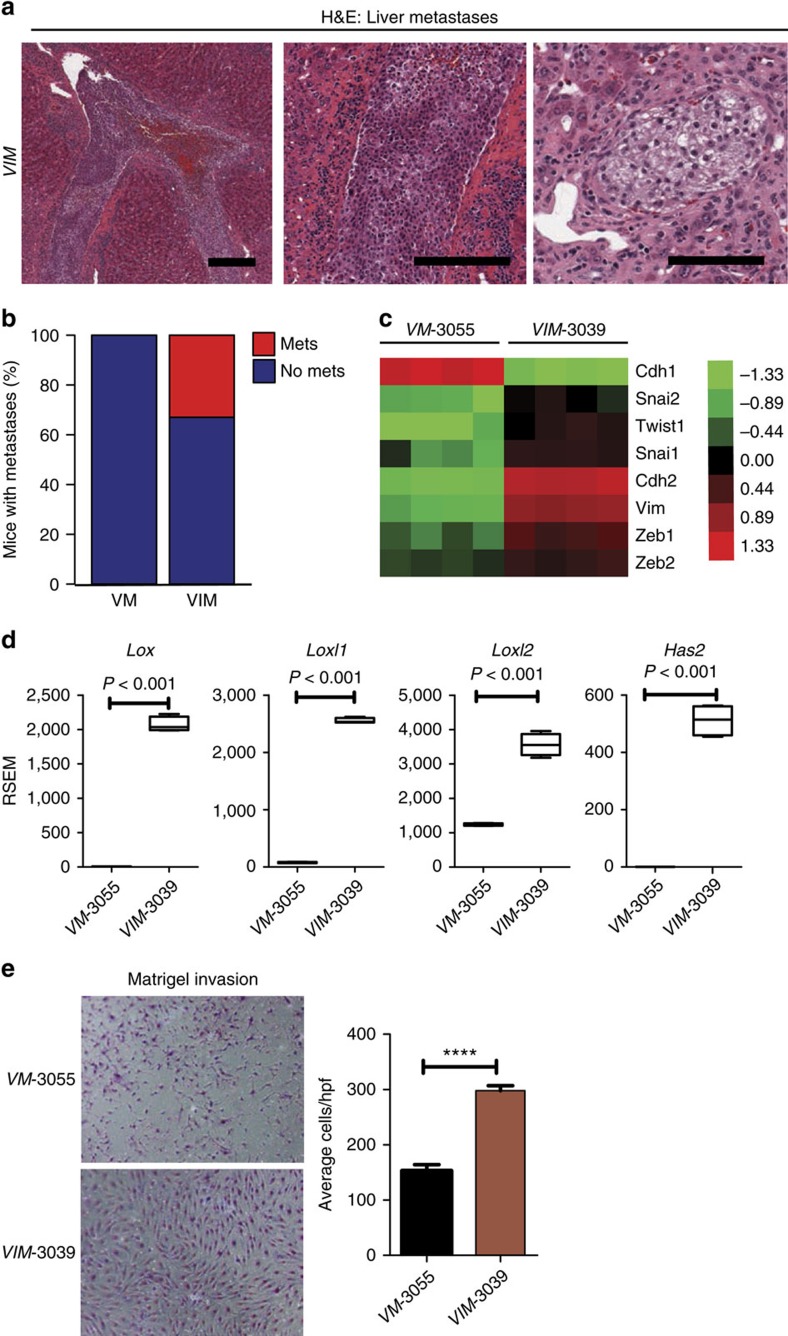
Combinatorial loss of *Vhl* and *Ink4a/Arf* with MYC activation promotes metastasis and activation of EMT genes. (**a**) A representative H&E stained liver sections from a *VIM* mouse shows metastasis to the liver parenchyma as well as intravascular mets. Higher power magnification reveals features resembling renal clear cell carcinomas within the metastatic lesions. (Scale bar, 100 μm). (**b**) Bar graph showing percentage of mice with metastases. (**c**) Heat map showing the expression patterns of EMT associated genes in *VM*-3055 and *VIM*-3039 cells. (**d**) Higher expression of genes involved in invasion and metastasis is observed in *VIM*-3039 cells compared to *VM*-3055 cells. (**e**) Matrigel invasion assays show increased invasion from *VIM*-3039 cells compared to *VM*-3055 cells. ****P<0.0001. (**d**,**e**) *P* values obtained from student *t*-test. Data are presented as mean ±s.e.m.

During necropsies of *VIM* mice, we noted that a number of mice had what appeared to be macroscopic metastases to the liver at a low frequency (Fig. 5a,b, and Table 1). Histologically, the liver metastases were either tubulo-solid or clear cell with cytologic features consistent with the primary renal tumour. Metastases were identified in the subcapsular area and within the parenchyma. Notably, vascular spaces were involved by tumour in some of the liver specimens (Fig. 5a). We did not see any macroscopic metastases to other organs such as the lung.

Given the metastatic phenotype seen in the *VIM* mice but not in the *VM* mice, we examined a panel of genes that characterize epithelial to mesenchymal transition (EMT) (*Cdh1* (E-cadherin), *Cdh2* (N-cadherin), *Vim* (vimentin)), as well as a panel of transcription factors well known to regulate EMT (*Snai1, Snai2, Zeb1, Zeb2, Twist1*) in the RNAseq data from our cell lines. *VIM-3039* cells demonstrated gene expression changes that were consistent with a state of EMT with decreased expression of E-cadherin (*Cdh1*) and increased expression of N-cadherin (*Cdh2*) and Vimentin (*Vim*) as well as upregulated expression of *Snai1, Snai2, Twist1, Zeb1* and *Zeb2* (Fig. 5c). In addition, we noted that a number of the most differentially expressed genes (Supplementary Data 4) are implicated in invasion and metastasis through remodelling of the extracellular matrix such as the lysyl oxidase family members *Lox, Loxl1, Loxl2* and hyaluronan synthase 2 (*Has2*) (Fig. 5d)^28^. Given these differences in gene expression, we examined the invasiveness of our *VM*-3055 and *VIM*-3039 cell lines *in vitro* in matrigel invasion assays. As predicted, *VIM-*3039 cells had significantly increased matrigel invasion (Fig. 5e). Therefore, *Ink4a/Arf* inactivation appears to facilitate EMT, invasion and metastases and the metastatic phenotype seen *in vivo* is recapitulated *in vitro*.

### VHL restoration has no effect on *VIM* cells

**Figure 6:**
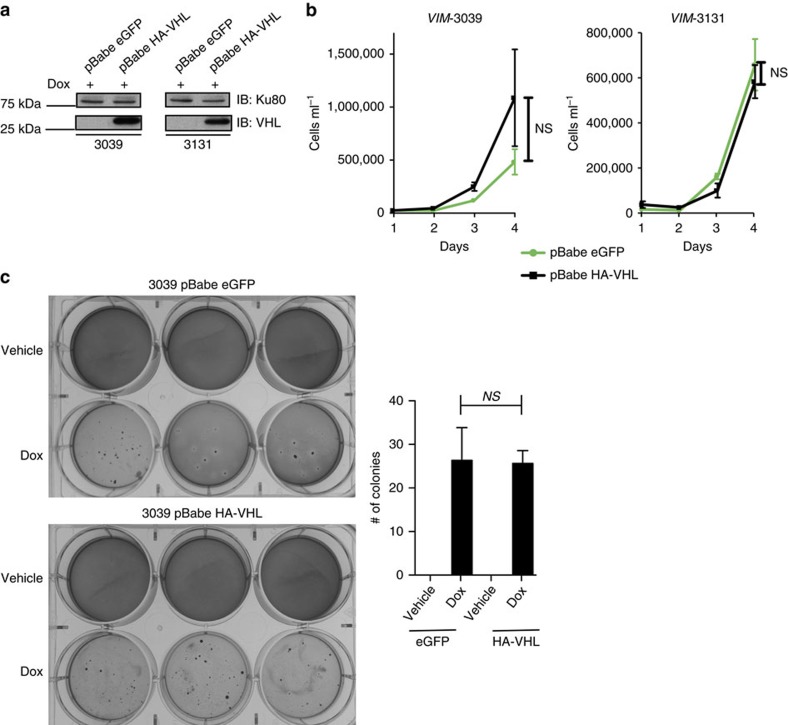
Restoration of VHL does not significantly reverse tumorigenic capacity of *VIM* cells. (**a**) Immunoblot of whole cell lysates from *VIM* cells infected with pBabe eGFP and pBabe HA-*VHL*. (**b**) Cell growth assay shows no significant reduction in cell number in *VIM* cells when *VHL* is expressed. Cells grown on Dox or vehicle were analysed in triplicate each day. (**c**) Soft agar assays show *VIM*-3039 cells form colonies in an anchorage independent manner despite the presence of *VHL*. (**b**,**c**) *P* values obtained from student *t*-test. Data are presented as mean ±s.e.m.

Previous work in human ccRCC cell lines has shown that VHL does not affect *in vitro* proliferation or anchorage independent growth^33^. To assess whether this was true in our *VIM* cell lines, we established isogenic *VIM* cells expressing eGFP or HA-VHL (Fig. 6a). As predicted, there were no VHL dependent differences in *in vitro* growth (Fig. 6b) or ability to form colonies in soft agar (Fig. 6c). These results are in keeping with those seen in human ccRCC cell lines. At this time we have not been able to reliably generate allograft tumours from our *VM* or *VIM* cell lines and therefore cannot interrogate whether VHL plays a tumour suppressor role *in vivo,* as previously shown with human RCC cell lines^33^. Therefore, whether our GEM models are overdriven remains a possibility.

## Discussion

The development of GEM models of cancer has had significant impact on functional genomics as well as preclinical development of novel therapies^34^. Indeed, robust GEMMs of non-small cell lung cancer^36^ and pancreatic ductal adenocarcinoma^37^ have led to an explosion of research into the biology underlying these diseases and facilitated the study of diverse fields such as biomarker discovery and investigations into the cell of origin of various cancers. Our own studies highlight the role of MYC in renal tumorigenesis and demonstrate that MYC activation is sufficient to generate papillary RCC and that when combined with *Vhl* and *Ink4a/Arf* inactivation results in bona fide clear cell renal cell carcinoma.

Development of robust GEM models of kidney cancer has been a long-sought after and elusive goal despite substantial efforts in this area. Multiple groups have now demonstrated that *Vhl* inactivation either in the germline or by conditional inactivation in the kidney results in only a mild increase in rate of renal cyst formation^26^,^38^,^39^,^40^,^41^. While the addition of secondary genetic events such as *Pten* or *Kif3a* loss appear to accelerate the cystic phenotype induced by *Vhl* inactivation, they do not result in frank neoplasia^42^,^43^. Combined inactivation of *Vhl* with *Trp53* does appear to induce renal tumours, which have clear cell changes but not bona fide clear cell RCC histology^44^. A recent report demonstrated that combined inactivation of *Vhl* with *Bap1* results in renal tumours with clear cell histology^45^. However, while inactivation of other tumour suppressor genes such as *Flcn*, *Tsc1* or *Fh* results in renal carcinomas^46^,^47^,^48^,^49^,^50^, they are rarely of the clear cell histologic subtype and they exhibit long latency, impacting their utility for routine investigation. Therefore, there remains a need for RCC models that are highly penetrant, relatively rapid and that display robust papillary or clear cell histology.

Schroff and colleagues recently reported the phenotype of kidney specific overexpression of c-MYC in mice^21^. They found robust development of renal tumours that were dependent on upregulated glutaminolysis. Histological and immunohistochemical analysis demonstrated that these tumours were most consistent with collecting duct carcinomas. Collecting duct carcinomas are thought to arise from the collecting ducts, which are embryologically derived from the metanephros and distinct from the nephron. In support of this, the immunophenotype, as well as response to therapy are closely associated with urothelial carcinomas (bladder cancer)^51^ and as such, they are treated with bladder cancer specific chemotherapy regimens such as MVAC (methotrexate, vinblastine, adriamycin, and cisplatin). Our studies also examined the phenotype of kidney specific overexpression of c-MYC. However, in contrast to Schroff and colleagues our *MYC* mice developed renal tumours with histology highly reminiscent of human papillary RCC. We hypothesize that these distinctions may be due to the relative differences in expression patterns of the promoters used to overexpress MYC (Schroff *et al*. use γ-glutamyl transpeptidase while our studies use Ksp-cadherin), as well as any potential differences in the timing of MYC activation.

Our mouse models of pRCC and ccRCC faithfully recapitulate the genomics of human RCC although the incidence of ccRCC tumours that have coincident *VHL* and *CDKN2A* inactivation, along with MYC activation is only ∼6%. The genomics of papillary and clear cell RCC implicate MYC as a potential oncogenic driver event^5^,^12^,^15^ and our studies firmly place MYC as playing an active role in their pathogenesis. *VHL* inactivation results in the stabilization of the alpha subunits of the hypoxia-inducible factor alpha (HIF) family of transcription factors of which HIF2α is thought to be a key oncogenic driver of ccRCC tumorigenesis, while emerging evidence suggests that HIF1α may be a tumour suppressor gene^3^,^8^,^10^,^11^,^52^,^53^. Past work examining the potential interaction between HIFα subunits and MYC suggest that while HIF1α disrupts MYC transcriptional activity, particularly of those genes involved in cell cycle progression, HIF2α actually facilitates MYC/MAX interactions^18^,^19^,^54^. It will be interesting to determine whether these HIF and MYC interactions occur *in vivo* in our *VM* and *VIM* mouse models and whether these oncogenic dependencies can be used as therapeutic vulnerabilities.

The *Ink4a/Arf* locus is best known as a cell autonomous barrier to cellular transformation through its negative regulation of cell cycle progression and its ability to promote cellular senescence^55^. Our studies demonstrate that *Ink4a/Arf* inactivation promotes liver metastases in an autochthonous ccRCC GEM model and that *Ink4a/Arf* loss is associated with gene expression patterns of EMT. Several past studies support that *Ink4a/Arf* may regulate EMT and metastasis. For example, in the mid 1990s, Allan Balmain and colleagues noted that p16 loss was a critical event in the regulation of an invasive spindle cell phenotype of mouse skin carcinomas, which today would likely be termed EMT^56^. In addition, a recent genome wide CRISPR screen in a cell line xenograft model identified *CDKN2A* as strongly associated with increased metastases^57^. At this time we hypothesize that *Ink4a/Arf* loss does not directly regulate EMT or metastases but that its loss is permissive for the emergence of clones that allow metastatic behaviour. Nonetheless, our studies bolster the notion that *Ink4a/Arf* loss is a metastatic driver event and demonstrate this association in autochthonous ccRCC GEM models.

In summary, we present GEM models of papillary and clear cell RCC that are faithful to the genomic events and recapitulate the histology seen in their respective human correlates. These pRCC and ccRCC GEM models should be a valuable contribution to the field of kidney cancer research and given their immune competent state should be invaluable in the development of immune based therapy.

## Methods

### GEM Models

Conditional Vhl knock-out mice (*Vhl*^*F/F*^)^26^, germline *Cdkn2a* (*Ink4a/Arf*) knockout mice (*Ink4a/Arf*^*−/−*^) (ref. 27), *KspCad-Cre*^*ERT2*^ (ref. 58), *KspCad-rtTA*^58^ and *Tet-O-Myc* mice^22^ have all been previously described. *Tet-O-Myc* was a generous gift from Dr Dean Felsher. Protocols for all animal experiments described were approved by the UNC-CH Institutional Animal Care and Use Committee.

### Reagents for *in vivo* studies

Conditional activation of cre recombinase was induced by oral administration of tamoxifen (Sigma T5648). Per ml of *in vivo* oral delivery, tamoxifen was formulated as: 100 mg of tamoxifen dissolved in 100 μl 100% EtOH, followed by the addition of 1 ml of sunflower oil to achieve 100 mg ml^−1^. The solution was sonicated in a water bath sonicator located at 4 °C for 10-s pulses. The solution was then placed in a water bath at 50 °C for 1 min until clear. Sonication and water bath steps were repeated until no particulates were noticeable. Animals were given 50 μl (5 mg per day) for three consecutive days by oral gavage. Tamoxifen oral solution was aliquoted and stored at −20 °C and brought to administrable solution by 50 °C water bath for no longer than 5 min. The Tet-ON conditional system was activated with doxycycline chow (Research Diets, Inc. C11300–2000 with 2,000 p.p.m. doxycycline) 7 days post oral tamoxifen administration.

### Primary tumour cell line generation and culture conditions

Primary renal GEMM tumours were excised and washed in a solution of Pen-Strep, PBS solution (1:1). In sterile conditions, primary tumours were cut into 2 × 2 mm fragments and dissociated in a gentleMACS C-Tube (Miltenyi Biotec) using the gentleMACs Dissociator (program: m_imp Tumor_02) in 5 ml of 1XDMEM, 10% FBS, 1% PenStrep. 100 μl of collagenase D/dispase II (Roche: 40 mg ml^−1^) was added to the tumour fragments and continuously inverted for 30 min at 37 °C. Fragments were then subjected to a second round of dissociation using the gentleMACS Dissociator (program: m_imp Tumor_03). A total of 5 ml of protein extraction buffer (PEB: buffer 0.5% FBS, 2 mM EDTA in PBS) was added to the dissociated fragments and resuspended by pipetting. The cell suspension was transferred to a 50 ml conical tube through a 40 μm nylon mesh sterile cell strainer (Fisher). An additional 20 ml of PEB buffer was added to the cell suspension and then centrifuged at 300*g* for 5 min. Supernatant was removed and cell pellet was resuspended in 6 ml of ‘conditioned media'^59^ containing 2 μg ml^−1^ doxycycline (Sigma) and placed in a 6 cm sterile cell culture plate.On reaching confluency, cells were split and cultured in 1XDMEM, 10% FBS, 1%PS containing 2 μg ml^−1^ doxycycline. *In vitro* doxycyline was dissolved in DMSO.

### Xenograft

Primary renal GEMM tumour cell lines were implanted subcutaneously into SCID mice flanks at 5 × 10^6^ cells in 200 μl of PBS containing 2 μg ml^−1^ doxycycline. To maintain expression of c-MYC, SCID mice harbouring xenografts were placed on doxycycline chow and withdrawn from doxycycline at the indicated times.

### Immunoblotting conditions

Cells were lysed in RIPA buffer complemented with Set I and Set II phosphatase inhibitors at 1 × (Calbiochem), and protease inhibitors at 1 × (Roche). Whole cell lysate concentration was determined with Bio-Rad Protein Assay Dye Reagent Concentrate (Bio-Rad). Proteins were resolved on SDS–PAGE gels and electrotransferred to nitrocellulose membranes, 0.2 μm (Bio-Rad). For detection of c-MYC protein, proteins were electrotransferred onto PVDF membranes (Millipore). Primary antibodies, c-Myc (1:1,000; SC-42), VHL (1:500; SC-5575), Ku80 (1:1,000; Cell Signaling #2,180), Vinculin-HRP (1:1,000; Cell Signaling #18799); SC: Santa Cruz Inc. (Supplementary Fig. 6).

### Cell viability assay

Cell viability in the context of the various culture conditions was measured by CellTiter-Glo Luminescent Cell Viability Assay (Promega) per manufacture's protocol. Cells were counted and in a 96 well opaque side/clear bottom cell culture plates (Corning). Luminescence measurements were captured using a Biotek Synergy 2 plate reader. Statistical significance was measured by student *t*-test.

### Soft agar assay

Anchorage independent growth was assayed following a standard soft agar assay protocol (bottom layer, 0.6% Difco Noble Agar (BD Biosciences) and top layer 0.4% Noble agar (Difco)). 200 μl of control media or media containing 2 μg ml^−1^ doxycycline was added to hydrate top agar every 72 h. A total of 5,000 cells were plated in each well of a tissue culture treated 6 well plate (Corning). Colonies were stained with crystal violet solution for 4 h and destained with H_2_O. Pictures were taken using a digital camera (Cannon). Treatment conditions were performed in triplicate. Statistical significance was measured by *t*-test.

### Mouse RNAseq

An equal number of cells (2 × 10^5^ cells per dish) were plated with or without Dox and collected for RNA 24 h later. RNA was isolated and purified for RNAseq analysis using the RNeasy Mini Kit (Qiagen) following manufacturer's protocol and eluted in water. A total of 200–1,000 ng of total RNA was used to prepare libraries with the TruSeq Stranded mRNA Sample Prep Kit (Illumina). Around 75b paired-end reads were sequenced on a NextSeq 500 Desktop Sequencer using a high output flow cell kit (Illumina), yielding an average of over 28 M reads per sample. QC-passed reads were aligned to the mouse reference genome (mm9) using MapSplice^60^. The alignment profile was determined by Picard Tools v1.64 (http://broadinstitute.github.io/picard/). Aligned reads were sorted and indexed using SAMtools and translated to transcriptome coordinates then filtered for indels, large inserts and zero mapping quality using UBU v1.0 (https://github.com/mozack/ubu). Transcript abundance estimates for each sample were performed using RSEM, an expectation-maximization algorithm (Li and Dewey, 2011) using the UCSC knownGene transcript and gene definitions^61^. Raw RSEM read counts for all RNAseq samples were normalized to the overall upper quartile^62^.

Gene set analysis was performed between two groups of samples by first ranking all genes by *t*-statistic between groups. A K–S test was performed to determine if the genes in each gene set from MSigDB were uniformly distributed among the ranked list. K–S test *P* values were corrected via B–H procedure with an overall FDR of 5%. ROC curves were then generated for each gene set using the ranked list of *t*-statistics, and the AUC for the top 10% of ranked genes was used to rank gene sets among those passing the K–S test.

### Analysis of human renal gene expression data from TCGA

GSE11151 data set was downloaded on Jan 27, 2015 from Gene Expression Omnibus (GEO) website. RNA Expression dataset was log2-transformed/median centred across pRCC and normal tissue samples. RNA expression dataset was analysed with the Gene Set Enrichment Analysis (GSEA) desktop platform.

TCGA KIRP dataset was downloaded on Feb 1, 2015 from Broad TCGA website. RNA Expression dataset was log2-transformed/median centred. RNA expression data set was analysed with the GSEA desktop platform.

TCGA KIRC data sets were downloaded on Feb 15, 2015 from Broad TCGA website. RNA Expression dataset was log2-transformed/median centred. RNA expression dataset was analysed with the cBioPortal Oncoprint function and on R platforms.

### Analysis of human copy number data from TCGA

Gene-level segmented DNA copy values for the TCGA KIRC cohort (*n*=528) were downloaded from the Broad Institute GDAC (https://gdac.broadinstitute.org/). The GISTIC2 analysis (Mermel, 2012) identified a statistically significant (q<0.25) region of amplification in chr8 (69506698-146364022). Mean gene-level DNA copy number measurements were computed and plotted by genomic position.

### Correlation analyses

The TCGA clear cell renal cell carcinoma (KIRC) and papillary renal cell carcinoma (KIRP) RNASeq expression datasets were downloaded through the Broad Institure pipeline (Broad Institute TCGA Genome Data Analysis Center (2016): Aggregate Analysis Features. Broad Institute of MIT and Harvard). Human homologues were identified for mouse genes from the Jackson Laboratory Mouse Genome Informatics database (http://www.informatics.jax.org/homology.shtml). When combining datasets, batch effects were adjusted for using the SVA (surrogate variable analysis) R package version 3.12.0. Centroids were derived by taking the median expression of each gene across samples in a designated sample cohort. Centroid similarity metrics were derived by calculating the Pearson correlation between centroids. Heatmap clustering was done using centred average linkage clustering on all expressed genes in the data set.

### Matrigel invasion assay

RCC GEMM cell lines were plated in serum-free medium for 24 h. A total of 50,000 cells were seeded into an 8-μm matrigel chamber (BD Biosciences) and placed in wells containing medium containing 10% FBS. Cells were allowed to invade for 24 h. Cells that had invaded through the matrigel were then stained with the Siemens Staining Kit according to manufacturer's directions. Matrigel wells were then placed on a glass slide and analysed and photographed using an Olympus IX51 microscope. Representative pictures were taken of each matrigel well quadrant, and the number of cells that had invaded into the matrigel was determined by counting the number of stained nuclei using ImageJ.

### Data availability

Raw RNA sequencing data are available from NCBI GEO under accession no. GSE97654.

## Additional information

**How to cite this article:** Bailey, S. T. *et al*. *MYC* activation cooperates with *Vhl* and *Ink4a/Arf* loss to induce clear cell renal cell carcinoma. *Nat. Commun.* **8,** 15770 doi: 10.1038/ncomms15770 (2017).

**Publisher's note:** Springer Nature remains neutral with regard to jurisdictional claims in published maps and institutional affiliations.

## Supplementary Material

Supplementary Information Supplementary Figures and Supplementary Table

Supplementary Data 1 Average DNA copy number of genes within the MCR of amplification on chr8.

Supplementary Data 2 Significantly enriched gene signatures within *VM*-3055 cells with or without Dox.

Supplementary Data 3 Significantly enriched gene signatures within *VIM*-3039 cells with or without Dox.

Supplementary Data 4 Differentially expressed gene list between *VIM*-3039 and *VM*-3055 cell lines.

## Figures and Tables

**Table 1:** Comparison of *VM* and *VIM* mice.

	***VM***	***VIM***
Median survival (weeks after Tam/Dox)	57	29.5
Cysts present	9/9 (100%)	6/6 (100%)
Kidney tumour present	6/9 (67%)	6/6 (100%)
Large kidney tumour (>3 mm)	2/9 (22%)	4/6 (67%)
Low grade	3/9 (33%)	1/6 (17%)
High grade	3/9 (33%)	5/6 (83%)
Necrosis present	1/9 (11%)	4/6 (67%)
Bona fide clear cell histology	0/9 (0%)	5/6 (83%)
Liver metastases	0/9 (0%)	2/6 (33%)
