# Supplementary material for: MYC activation cooperates with Vhl and Ink4a/Arf loss to induce clear cell renal cell carcinoma
Source: Nat Commun. 2017 Jun 8;8:15770. doi: 10.1038/ncomms15770 (PMC5472759; doi:10.1038/ncomms15770)
Supplement: Supplementary Information — Supplementary Figures and Supplementary Table [file ncomms15770-s1.pdf]

a

pleomorphism

MYC

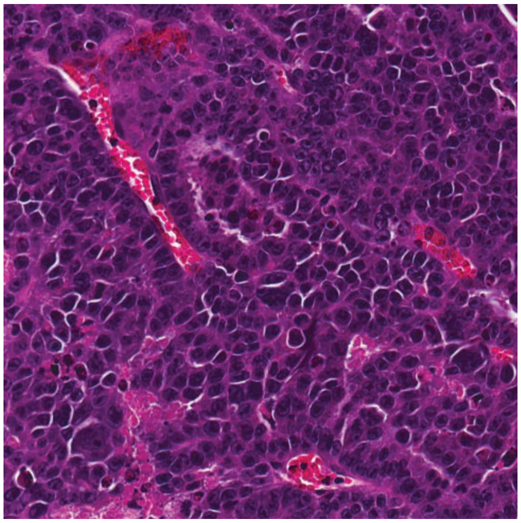

b

PID_MYC_PATHWAY

DANG_MYC TARGETS_UP

PID_MYC ACTIV_PATHWAY

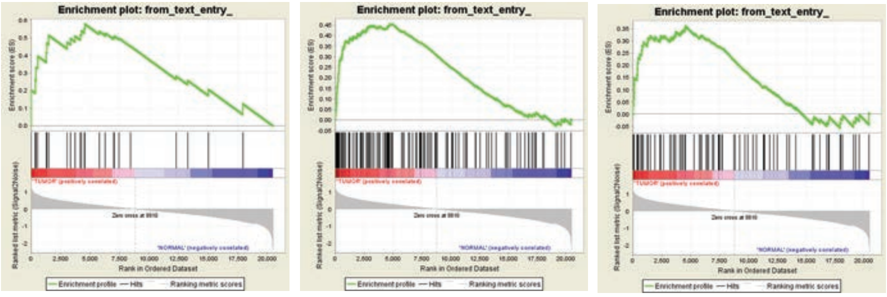

ES: 0.58
p value: 0.021

ES: 0.45
p value: 0.24

ES: 0.36
p value: 0.20

Supplementary Figure 1. MYC activation in the kidney results in histological abnormalities. (a) H&E staining of a representative kidney section from a *MYC* mouse reveals pleomorphism. **(b)** GSEA enrichment plots showing papillary tumors from TCGA KIRP are enriched for gene sets representing MYC activation relative to normal kidney.

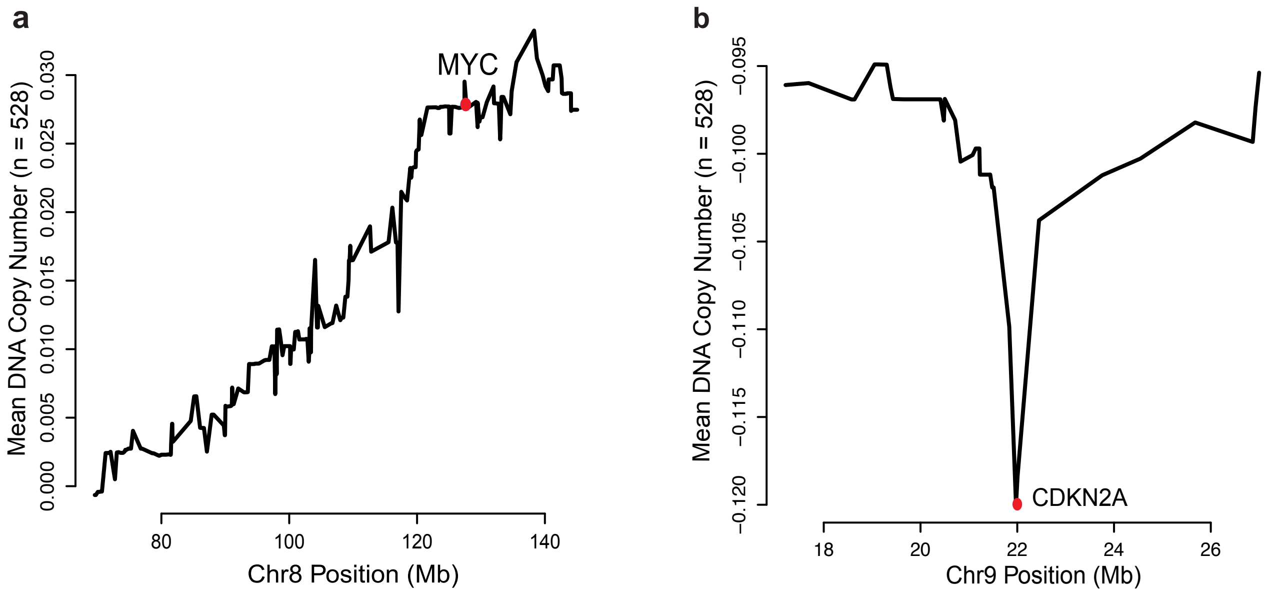

Supplementary Figure 2. GISTIC copy number by gene. (a) GISTIC2 analysis on TCGA KIRC cohort of patients showing average DNA copy number measurements within region of amplification in chr8 for MYC and **(b)** region of deletion of chr9 for CDKN2A.

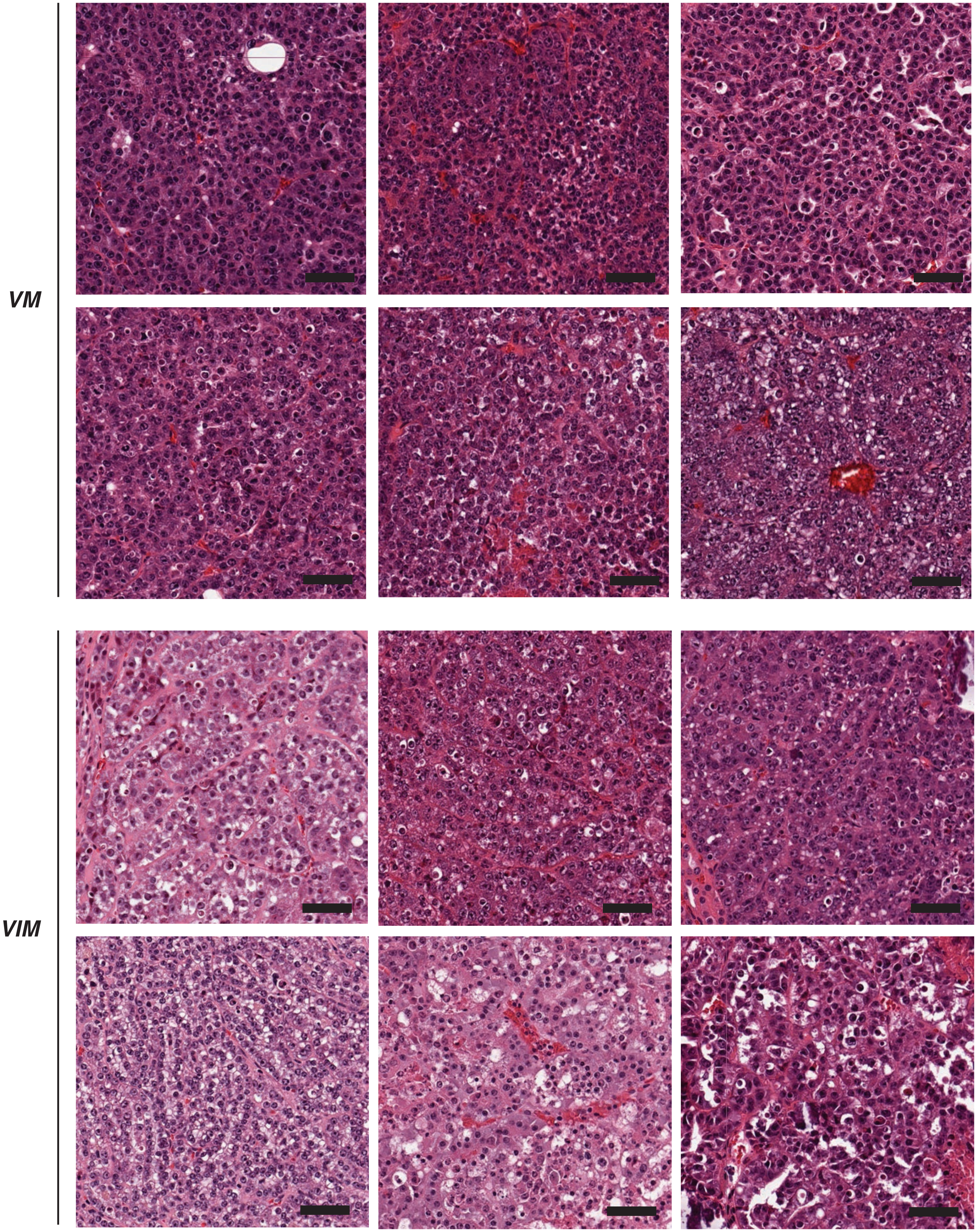

Supplementary Figure 3. Representative H&E images of VM and VIM mouse tumors. (scale bar = 50um)

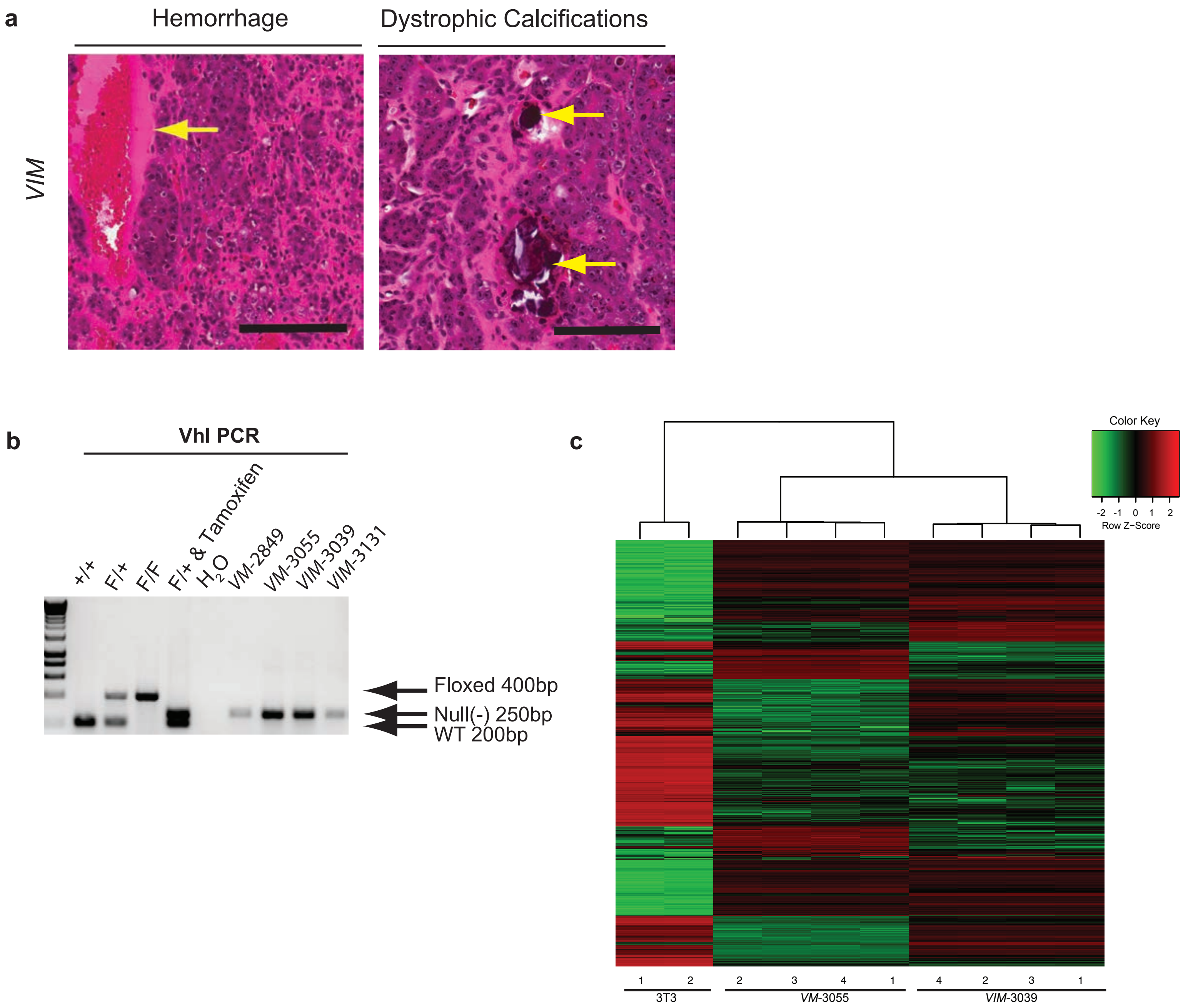

Supplementary Figure 4. ***VM* and *VIM* mice have histological abnormalities in their kidneys. (a)** H&E staining of a representative kidney section from a *VM* tumor. Arrows point to areas of hemorrhage and dystrophic calcifications. **(b)** PCR verfication of Vhl deletion in the *VM* and *VIM* cell lines. **(c)** Heat map showing distinct expression patterns between 3T3 fibroblasts and *VM*-3055 and *VIM*-3039 cell lines.

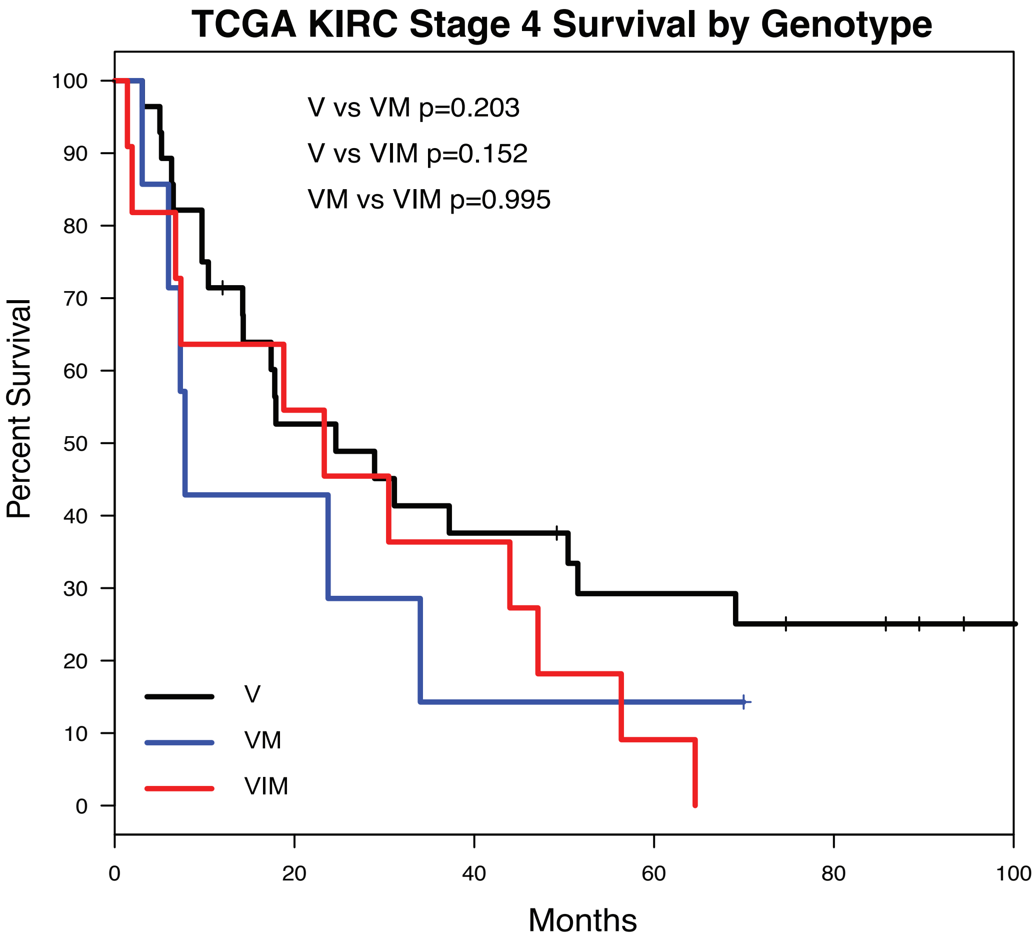

Supplementary Figure 5. No difference in survival among stage IV patients. Kaplan-Meier survival curve of TCGA KIRC stage IV KIRC patients with V, VM, or VIM genotype. V (n=28), VM (n=7), VIM (n=11). Log rank V vs VM p=0.203, V vs VIM p=0.152, VM vs VIM p=0.995.

A

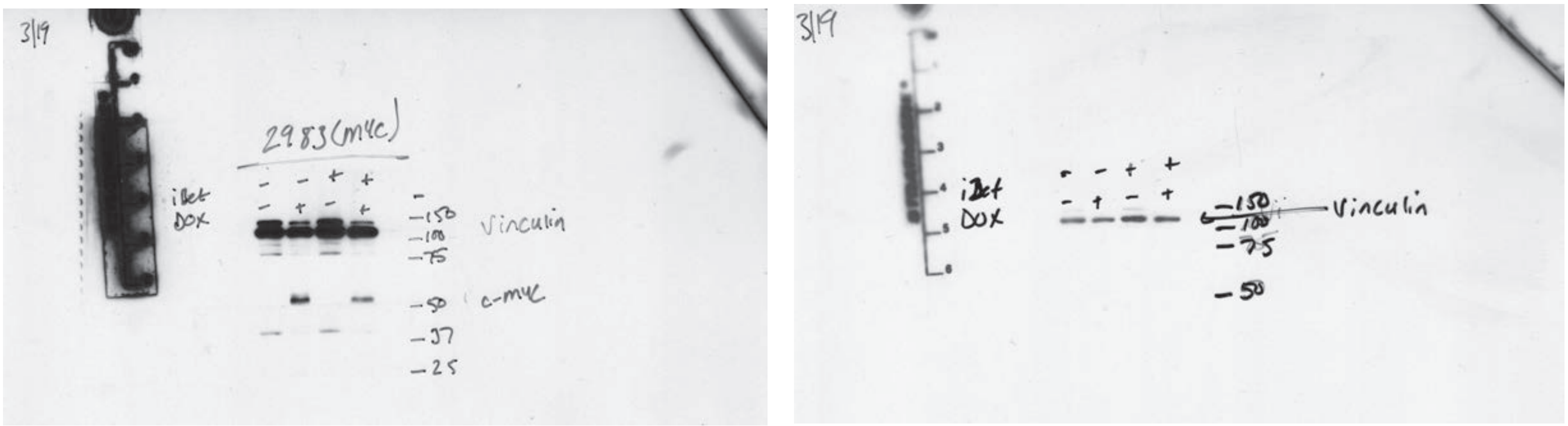

B

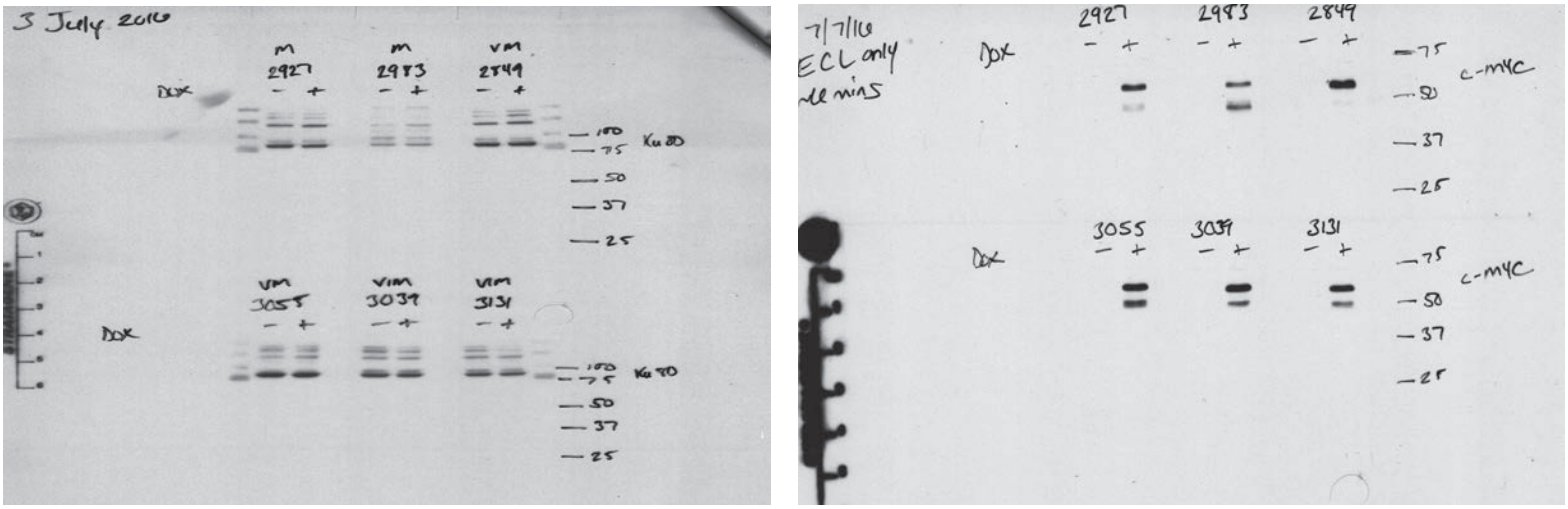

C

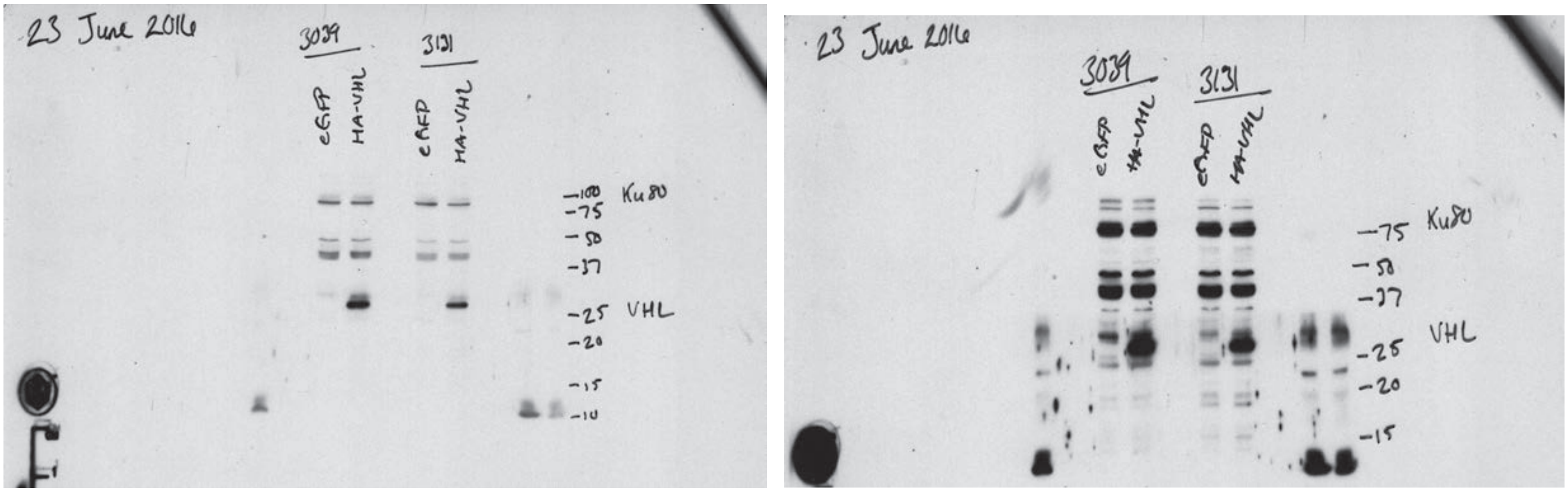

Supplementary Figure 6. Un-cropped scans of western blot membranes. (a) *MYC*-2983 blot (Figure 1E). **(b)** *MYC*-2927 blot (Figure 1E, *VM* and *VIM* cell lines (Figure 3A). **(c)** *VIM*-3039 and *VIM*-3131 pBabe eGFP and pBabe HA-VHL (Figure 6A).

Supplemental Table 1. Summary of RCC GEM models

Abbreviation	Genotype	Functional Effect
MYC	*Ksp-rtTA; Tet-O-MYC*	• Dox inducible *MYC* expression
V	*KspCreERT2;* $Vhl^{F/F}$	• conditional inactivation of *Vhl*
VM	*KspCreERT2;* $Vhl^{F/F}$*; Ksp-rtTA; Tet-O-Myc*	• conditional inactivation of *Vhl*<br>• Dox inducible *MYC* expression
VIM	*KspCreERT2;* $Vhl^{F/F}$*;* $Ink/Arf^{-/-}$*; Ksp-rtTA; Tet-O-Myc*	• conditional inactivation of *Vhl*<br>• germline $Ink/Arf^{-/-}$<br>• Dox inducible *MYC* expression
